# Intermittent Explosive Disorder amongst Women in Conflict Affected Timor-Leste: Associations with Human Rights Trauma, Ongoing Violence, Poverty, and Injustice

**DOI:** 10.1371/journal.pone.0069207

**Published:** 2013-08-07

**Authors:** Susan Rees, Derrick Silove, Teresa Verdial, Natalino Tam, Elisa Savio, Zulmira Fonseca, Rosamund Thorpe, Belinda Liddell, Anthony Zwi, Kuowei Tay, Robert Brooks, Zachary Steel

**Affiliations:** 1 Psychiatry Research and Teaching Unit, University of New South Wales, Level 2 Mental Health Centre, The Liverpool Hospital, Sydney, Australia; 2 Alola Foundation, Dili, Timor-Leste; 3 Social Work and Community Welfare, School of Arts and Social Sciences, James Cook University, Townsville, Australia; 4 School of Psychology, University of New South Wales, Sydney, Australia; 5 School of Social Sciences, University of New South Wales, Sydney, Australia; University of Medicine & Dentistry of NJ – New Jersey Medical School, United States of America

## Abstract

**Introduction:**

Women in conflict-affected countries are at risk of mental disorders such as posttraumatic stress disorder and depression. No studies have investigated the association between experiences of abuse and injustice and explosive anger amongst women in these settings, and the impact of anger on women's health, family relationships and ability to participate in development.

**Methods:**

A mixed methods study including an epidemiological survey (n = 1513, 92.6% response) and qualitative interviews (n = 77) was conducted in Timor-Leste. The indices measured included Intermittent Explosive Disorder, posttraumatic stress disorder; severe distress; days out of role (the number of days that the person was unable to undertake normal activities); gender-specific trauma; conflict/violence; poverty; and preoccupations with injustice.

**Results:**

Women with Intermittent Explosive Disorder (n = 184, 12.2%) were more disabled than those without the disorder (for >5 days out of role, 40.8% versus 31.5%, X^2^_(2)_  = 12.93 p = 0.0016). Multivariable associations with Intermittent Explosive Disorder, controlling for the presence of PTSD, psychological distress and other predictors in the model, included the sense of being sick (OR 1.73; 95% CI 1.08–2.77); victimization as a result of helping the resistance movement (OR 2.33, 95% CI 1.48–3.68); war-related trauma specific to being a woman (OR 1.95, 95%, CI 1.09–3.50); ongoing family violence and community conflict (OR 1.88, 95% CI 1.27–2.77); extreme poverty (OR 1.23, 95%, CI 1.08–1.39); and distressing preoccupations with injustice (relating to 2/3 historical periods, OR 2.10, 95% CI 1.35–3.28). In the qualitative study, women elaborated on the determinants of anger and its impact on their health, family and community functioning, child-rearing, and capacity to engage in development. Women reflected on the strategies that might help them overcome their anger.

**Conclusions:**

Intermittent Explosive Disorder is prevalent and disabling amongst women in conflict-affected Timor-Leste, impacting on their health, child-rearing and ability to participate fully in socio-economic development.

## Introduction

Mental disorders such as posttraumatic stress disorder (PTSD) and depression are prevalent in conflict-affected countries, but there has been little focus on other reactions to extreme forms of injustice such as explosive anger [1]. Women may be at special risk of explosive anger because of the particular forms of human rights violations and hardships they suffer in these settings, including exposure to gender-specific abuses, and bearing a disproportionate burden in relation to poverty and child-rearing [2], [3]. Explosive anger in turn may impact on women's health and their interpersonal interactions in the family and the wider community. At a more general level it is important to determine whether high levels of anger amongst women undermine the critical role they play in achieving social stabilization and development in the aftermath of conflict [3]–[6].

Surprisingly, little attention has been given to the study of pathological forms of anger in mental health research. Recently however, studies have focused on the psychiatric category of Intermittent Explosive Disorder (IED) which is characterized by the Diagnostic and Statistical Manual edition 4 of the American Psychiatric Association as comprising two components: repeated attacks of extreme anger in response to minor environmental triggers and associated acts of physical aggression involving persons or property.

The small number of epidemiological studies examining IED have indicated that the disorder is prevalent in developed societies such as the US and that it affects both men and women [7]. In a previous study conducted in the same setting as the present inquiry in Timor-Leste, we examined a broader definition of explosive anger, applying a culturally-adapted measure to assess the subjective experience of anger rather than explicit acts of aggression [2]. Our study recorded a high prevalence of explosive anger, with the rate for women (41%) exceeding that for men (38%) [2]. The study suggested a link between traumatic experiences associated with past human rights abuses and the emergence of anger, consistent with prevailing theory and early observations from the small number of studies conducted in other countries [2], [3], [8], [9]. The present study aimed to advance these preliminary findings by applying a strictly defined set of criteria for assessing IED, using a mixed methods approach to determine the antecedents and consequences of explosive anger amongst women in Timor-Leste.

In undertaking studies on anger amongst women, it is important to acknowledge that the reaction may be a normative response to legitimate grievances, motivating women to seek redress [10]. Nevertheless, if uncontrollable forms of explosive anger are damaging to the health and well-being of women, impacting adversely on their child-rearing abilities and capacity to participate in development, then it is vital to ensure that these adverse psychological outcomes of mass conflict are properly documented [10].

The history of conflict in Timor-Leste exemplifies the conditions that might be expected to precipitate and perpetuate extreme forms of anger amongst women. During the prolonged war of resistance against the Indonesian occupation (1975–1999), women were subjected to politically motivated rape, forced marriages, chemical sterilization, removal of children and traumatic loss of husbands and other family members [11]–[13]. Further abuses and deaths occurred on a large scale during the humanitarian emergency that occurred in 1999 in which Indonesian-supported militia destroyed 80% of the infrastructure of the country and displaced the majority of the population. In 2006, a period of internal conflict occurred, resulting in violence, destruction of property and displacement of communities. The villages in our study were directly impacted by that conflict.

Poverty continues to present a major challenge to large-sectors of the community, impacting disproportionately on women. Since gaining independence in 2002, Timor-Leste has remained one of the poorest nations in the world, with 23% of the population of 1.2 million being under-nourished [14], [15] Women have higher rates of malnutrition and substantially lower levels of literacy and numeracy than men [14].

In the present mixed methods study we hypothesized that IED would be prevalent amongst women in Timor-Leste and linked to past and contemporary gender-related abuses, ongoing violence, extreme poverty, and distressing preoccupations with past and current injustices. The qualitative component of the study aimed to elaborate on women's personal experiences of explosive anger and its impact on their health, child-rearing and capacity to participate in development activities. We also assessed women's perspectives about the possible strategies that might assist them to overcome anger.

## Methods

### Overview

Our mixed methods approach included an epidemiological survey and a qualitative component, the latter facilitating a safe exploration of gender-sensitive issues in a setting where cultural and religious factors might constrain disclosure of personal information [16], [17]. The qualitative component was undertaken according to the Consolidated Criteria for Reporting Qualitative Research (COREQ) checklist [18]. We adopted a theoretical perspective of pragmatism in which quantitative data are enriched by qualitative research in a manner that gives each approach equal weighting [19]–[21]. The two key principles of the pragmatic approach that are relevant to the present research are: 1.*Complementarity*, where the quantitative and qualitative methods are used to address different facets of the subject matter under investigation; in this instance, we used quantitative data to estimate the prevalence of IED and its association with risk factors, and the qualitative data to provide rich accounts of women's personal perspectives regarding the significance of these issues and the factors that may prevent or mitigate anger; and 2. *Confirmation*, where the results of two methods either converge or reveal dissonance or ambiguity [21].

Interviews were undertaken from May, 2010 to November, 2011, in participants' homes, lasting up to one hour for the survey, and one hour for the qualitative interview. Qualitative and quantitative interviews were completed on separate days for those participating in both arms of the study. We applied strict provisions of confidentiality and privacy according to World Health Organization guidelines [22].

### Partnerships and Ethics

The study was undertaken in partnership with the Alola Foundation, the largest women's NGO in Timor Leste. The Human Ethics Committee of the University of New South Wales and the Timor-Leste Ministry of Health approved the study. The information sheet regarding the study objectives and the involvement of participants was read verbally to each potential participant. We requested verbal consent from participants because of low rates of literacy, and possible anxiety associated with signing of forms. Participants provide their verbal informed consent to participate in the presence of a respected Timorese person and the interview staff. The interviewer signed the consent form stating that the participant had understood the information sheet detailing the study and had voluntarily agreed to participate. The process for verbal consent to participate was approved by the University of New South Wales Human Ethics Committee and the Timor-Leste Ministry of Health. We followed the World Health Organization protocol for conducting research that involves questions relating to gender-based violence [22]. To avoid the risk of participants experiencing retaliatory violence from husbands, the quantitative survey was presented as a mental health survey, and the qualitative study as focusing on family harmony. Because some questions referred to experiencing difficult emotions, the health professionals in our team were trained to be able to respond quickly to anyone experiencing distress, providing immediate support and if required or requested, prompt referrals were made to women's support agencies and/or community mental health services with which the team had close working relationships.

First we describe the quantitative survey and then the qualitative process.

### Quantitative Survey

We conducted a total household survey of adults, 18-years and older, residing in two villages, one urban, the other rural, the latter situated approximately one hour's drive from Dili, the capital. We were able to ensure complete coverage of dwellings by using GPS coordinates and maps generated by the National Directorate of Statistics who oversee the national census.

#### Personnel and Training

An Australian Project Director and a Timorese in-country manager supervised 18 Timorese field workers with prior survey experience and/or public health degrees. The field staff received two weeks' training followed by two months of field testing and questionnaire piloting in areas separate from the sites of the main study. Pairs of interviewers were required to achieve a consistent 100 percent level of inter-rater reliability on key diagnostic measures. The survey was conducted between June, 2010 and October, 2011.

#### Symptom measures

The community measure of IED was tested and modified serially during piloting to ensure its comprehensibility in the local language, Tetun. Given the high rates of comorbidity found for IED in other settings, we included The Harvard Trauma Questionnaire (HTQ) [23] for PTSD and the Kessler-10 scale (K10) [24] as a measure of severe distress that predominantly includes symptoms of depression but that also provides a general index of mental disorder [24]. A convergence study compared these measures with the relevant categories (IED, PTSD, major depressive episode) of the Structured Clinical Interview for the Diagnostic and Statistical Manual [7] applied in a blinded manner by experienced psychologists. There was a high level of concordance for all measures: Area Under the Curve for IED 0.90 (95% CI: 0.83–0.98), for PTSD 0.824 (95% CI: 0.705–0.943) and for severe distress measured by the K10 0.792 (95% C.I.: 0.67–0.914) (full data will be provided on request).

#### Gender-specific trauma and daily stresses

Community leaders indicated that it was culturally unacceptable to ask women specific questions about gender-based violence such as rape in the context of a door-to-door survey. We therefore piloted and included an item inquiring into ‘trauma suffered specifically because you are a woman.’ We also inquired whether women had suffered because they had helped the armed resistance, a common occurrence during the Indonesian occupation.

A list of daily living difficulties was established based on extensive community consultation, yielding two indices, each based on the sum of 6 items (scored 1 = yes, 0 = no): *poverty* comprised items inquiring into lack of money for basic needs, for school fees, and to meet traditional family commitments including the customary practice known as ‘lia,’ an obligation to contribute money or possessions to extended family; poor shelter; unemployment; and having to forego food to feed the family; *family/community conflict and violence* comprised items inquiring into conflict/violence in relation to spouse, children, and extended family; worry about youth violence; ongoing community tensions; and fears of future conflict in the society.

To assess distressing preoccupations with injustice, respondents were asked to identify and describe the worst human rights violation/injustice they had experienced during three defined historical periods, the Indonesian occupation, the period of internal conflict and currently. Participants who recorded an event were then asked whether they continued to have distressing thoughts about the injustice associated with these experiences (present  = 1; absent  = 0 for each time period). The sum of the items for each of the three periods provided the composite index of distressing preoccupations with injustice (0, 1, 2/3). The second and third categories (2/3) were combined because of low numbers endorsing 3 epochs.

We assessed disability during the past month according to standard items used in the World Mental Health Survey for the number of days that respondents were incapacitated or had to reduce work/their normal activities because of ill health [25].

#### Statistical analyses

Tetrachoric correlations were used to test for comorbidity [26]. Multiple logistic regression analysis controlling for the presence of PTSD and depression examined for associations between IED and socio-demographic characteristics (age in years <25, 25–34, 35–54, 55+), rural/urban residency, education: none/some, primary, secondary, post-school), indices of the gender-related trauma, poverty, ongoing conflict and distressing preoccupations with injustice. We used SAS software (V9.2; SAS Institute Inc., Cary NC, USA 2002–2008), controlling for clustering by household with the *surveylogistic* procedure. Tests of association are reported as odds ratios (ORs) with 95% Confidence Intervals (CIs), the level of significance set at *P*<.05. A c-statistic (Area Under the Curve) of 0.8 indicates a high level of association for the overall model.

### Qualitative Sample

Seventy-seven women were sampled sequentially from the database, oversampling those with IED (n = 64). Qualitative interviews and data analysis were undertaken iteratively, with sampling ceasing when further interviews produced a high level of informational redundancy [27]. In this case, we determined sampling to be adequate when we had obtained sufficient, rich, case-oriented data to answer deductively our research questions. In addition, we wanted a sample size with adequate breadth for potential generalizability of the findings to other groups of conflict-affected women [27], [28].

### Qualitative procedure

Phase 1 of the qualitative study involved in-depth interviews with 19 Timorese women, the domains explored being informed by the existing literature (non-peer reviewed reports and peer-reviewed publications) pertaining to the psychosocial conditions of women in Timor-Leste, mental health data from our past surveys and relevant areas of conflict theory [12], [13], [29]. Interviews were discussion-based, with interviewers using reflecting, prompting and clarifying techniques. Interview responses were written rather than audio-taped, following local advice that electronic recordings might be experienced as intimidating amongst a population that had been exposed to a prolonged repression.

Phase 2 (involving 58 participants) included a semi-structured interview of living difficulties compiled from the data gathered in Phase 1. Women were asked to expand on whether and how each stressor generated anger and on the manifestations of anger they experienced (physical, psychological, interpersonal, within the family, in the wider social sphere). For example, the questions included “Why do these kinds of issues make you angry” and “What are some things that happen to women when they are angry?” Women were also asked to reflect on the factors that might improve anger. Participants were asked “What could help women to be less angry?”

Phase III involved a focus group with workers from all non-government organizations in Timor-Leste working specifically with women, including in the domestic violence and mental health sectors. Data generated from the field interviews were discussed, clarified and checked by way of feedback from this professional sample.

#### Personnel and Training

Four Timorese women with prior qualitative research experience employed in the Maternal and Child Health unit of the Alola Foundation conducted the qualitative research under the supervision of an Australian Project Director and a Timorese in-country manager. The interviewers received two weeks’ training followed by field testing of interviews.

#### Mixed Methods Analysis

Two raters independently coded the raw data and minor differences were reconciled by consultation. We used QSR Nvivo 9 [30] for data management and analysis in order to examine dominant and possible dissonant themes and their inter-relationships. Triangulation of the key sources of data [31] was accomplished by using the quantitative data as the scaffolding on which the qualitative data were applied to build a more comprehensive picture, with the findings being cross-checked with NGO personnel in the focus groups [32]. Qualitative data are presented here with reference to either the rural or urban location of the participant if it is relevant. We have not included the ages of the women quoted to protect their identities. All interviews presented in this paper are from different participants.

## Results

The survey of 2964 persons included 1513 women (response rate for women  = 92.6%). There was a wide range of ages as reported in Table 1. 62.3% or women were rural residents, and the majority (68.9%) was married. Approximately half (55.3%) had no or only some primary education. The number of children per household ranged from 1 to 10. Full-time employment (48%) largely involved subsistence farming supplemented by a small shop or stall selling produce; 27.9% of women were subsistence farmers with no personal source of cash income and 24.1% were unemployed.

**Table 1:** Associations of socio-demographic factors, trauma, conflict, poverty and injustice with Intermittent Explosive Disorder amongst Timorese women.

		Women		IED		Adjusted ORs for IED		
		N	Col%	n	row%	OR 1	95%L	95%U
		1513	100	184	12.2			
Location								
	Rural	942	62.3	107	11.4	1.00	(Ref)	
	Urban	571	37.7	77	13.5	2.30	1.55	3.42
Age groups								
	<25	361	23.9	32	8.9	1.00	(Ref)	
	25–34	496	32.8	61	12.3	0.98	0.59	1.64
	35–54	447	29.5	66	14.8	0.70	0.41	1.20
	55+	209	13.8	25	12	0.54	0.26	1.09
Educational level								
	None to primary school	836	55.3	111	13.3	1.00	(Ref)	
	Secondary school or more	677	44.7	73	10.8	0.80	0.53	1.20
Marital status								
	Single/widowed/other	471	31.1	47	10.0	1.00	(Ref)	
	Married	1042	68.9	137	13.1	1.19	0.78	1.83
Suffered because helped resistance								
	No	1277	84.4	109	8.5	1.00	(Ref)	
	Yes	236	15.6	75	31.8	2.33	1.48	3.68
Suffered serious trauma as a woman								
	Yes	1406	92.9	143	10.2	1.00	(Ref)	
	No	107	7.1	41	38.3	1.95	1.09	3.50
Sickness (DS1)								
	Yes	1340	88.6	140	10.4	1.00	(Ref)	
	No	173	11.4	44	25.4	1.73	1.08	2.77
Severe poverty index (mean count,sd)		1.80 (1.64)		2.45 (1.88)		1.23	1.08	1.39
Family and Community conflict index (mean count,sd)		0.52 (0.82)		0.91 (0.93)		1.88	1.27	2.77
Distressing preoccupations with injustice 2								
	None	886	58.6	63	7.1	1.00	(Ref)	
	1 historical period	322	21.3	50	15.5	1.68	1.07	2.63
	≥2 historical periods	305	20.2	71	23.3	2.10	1.35	3.28

1 Odds Ratios adjusted for presence of PTSD, psychological distress and all other variables in the model.

2 3 historical periods: Indonesian occupation; Independence period.

Model c-statistic (Area Under the Curve)  = 0.799.

### Prevalence of IED and comorbidity

The rate of IED for women (184, 12.2%) was double that for men (n = 96, 6.6%). IED amongst women showed moderate levels of co-morbidity with PTSD (tetrachoric correlation coefficient: 0.36, 95% CI 0.26–0.46) and severe distress (0.41, 95% CI 0.31–0.51), with 40.8% (n = 75) of women having IED alone. The qualitative data indicated that explosive episodes commonly were associated with physical symptoms such as shaking and headaches.

*‘My anger happens one to two times a month. I feel shake on my hand, my body, dizzy and sometimes I smash things in the house. It mostly happens when the children provoke me.’*

### Factors associated with IED

Table 1 shows the results of the multiple logistic regression analysis. The c-statistic of .80 indicates that the combination of variables included in the model had a high level of association with IED.

#### Socio-demographic factors and education

Urban residency was the only socio-demographic characteristic associated with IED (OR 2.03, 95% CI: 1.55–3.42). In the qualitative interviews, urban women reported high levels of frustration associated with lack of educational and employment opportunities and being confined to traditional gender roles. *‘Women can change their behavior from being angry if they have job. In the future, we have to prepare our minds. If we have weak minds then the country will become weak too.’*

#### Sickness, disability and health care

Women with IED were more likely to report “feeling sick” (OR 1.73; 95% CI 1.08–2.77). A separate analysis controlling for socio-demographic variables showed that women with IED were more likely to experience 5 or more days out of role (a period when they were unable to undertake normal activities) in the past month due to ill-health (75, 40.8% with IED versus 418, 31,5% without IED) (X^2^_(2)_  = 12.93 p = 0.0016). In the qualitative data, women with IED commonly reported difficulties caring for family and working around the house. They attended clinics for symptoms of anger but were dissatisfied with the treatment: *‘I went to clinic and met the doctor for my anger but it did not make any change. I want to see good doctor but no money.’*

More generally, women expressed dissatisfaction with the availability of health care for themselves and their children.

#### Conflict and abuse

Victimization arising from helping the resistance (OR 2.33, 95% CI 1.48–3.68), experiencing trauma as a result of being a women (OR 1.95, 95%, CI 1.09–3.50) and the index of ongoing family and community conflict (OR 1.88, 95% CI 1.27–2.77) all were associated with IED in the multivariable analysis. Qualitatively, women recorded the risks they had faced of being raped or killed when they transported food, weapons and messages to resistance fighters during the war.

Women also described how prevailing patriarchal values, unemployment and mental illness in husbands combined to generate domestic violence. *‘My husband is ‘pontu’ (has a mental illness) and we don’t have a good relationship. After we came back from the mountain (as refugees) from the crisis in 2006 he started not being good to me. He always hit me every time he gets drunk.’*

Punishment of children and tensions with neighbours were closely inter-related. Women reported that their own children were subjected to inappropriate punishment by neighbours, or conversely, outsiders interfered with parents physically punishing their own children:

*‘My neighbors always do this (punish) to my children. Many times I have argued and fight with them just because of this bad attitude. I have to fight with them so I could feel calm. If I don’t express my anger, I will feel shake on my hand and body, dizzy and sometimes unconscious.’*

#### Poverty

The index of severe poverty was associated with IED (OR 1.23, 95%, CI 1.08–1.39). The qualitative data revealed that the stressors of poverty were closely associated with the perception of having too many children to care for adequately.

*‘I have too many children, ten of them. It makes my headache to look after them. Beside that the children are also very naughty. They make me talking a lot, and angry.’*

Qualitative interviews indicated that the loss of family and mentors in the conflict meant that women had insufficient parenting support in the post-conflict period. Not being able to manage children was a trigger of anger which in turn was enacted on the children, often leading to excessive and harmful punishment.

*‘For myself, if the children naughty I have to hit them then I can feel calm. Other women maybe they tell bad words, hit or threat the children badly.’*

‘Lia’, a system of customary financial and material obligations to extended family added to the pressures of daily life. *‘If we have lia, I feel very angry because our relatives will force us to contribute a lot of money or animals like goats or buffalos but if we don’t do that they will tell us badly.’*

#### Injustice

The index of distressing preoccupations with past and ongoing injustice was associated with IED (for 1 historical period, OR 1.68, 95% CI 1.07–2.63, for two or three periods, OR 2.10, 95% CI 1.35–3.28).

*‘(My) family was lost in the war in 1975, 1999 and 2006. One of my brothers died in the war in 1975. When I think back to this incident, I feel very angry. We lost our people and properties in the war. We have informed this many times to the government but no response. We are the victims of high level’s neglect. We suffer a lot.’*

The qualitative interviews revealed that distressing preoccupations with injustice were compounded by women's dissatisfaction with their contemporary lives, in particular, not being able to access education or employment, and being encumbered by the burden of daily survival.

### Factors that might mitigate or ameliorate anger

**Figure 1:**
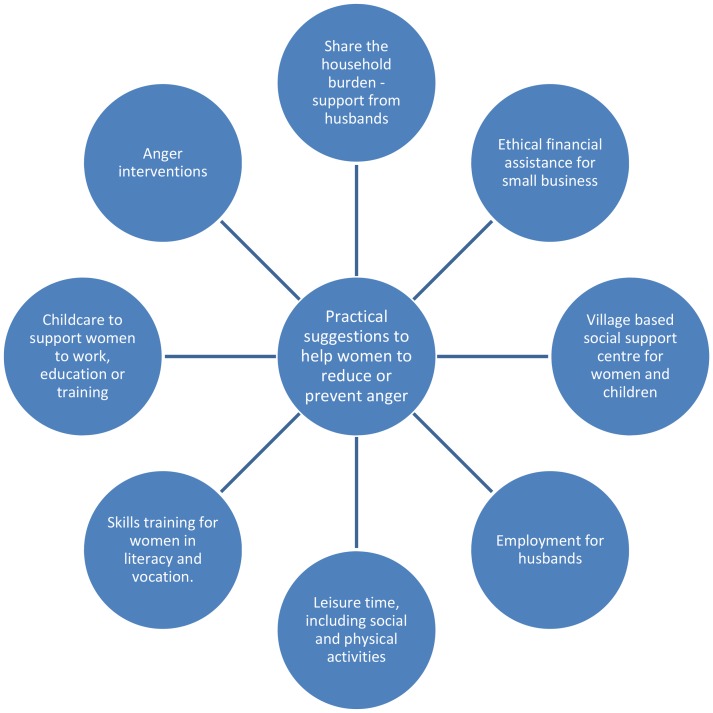
Practical suggestions women provided to ameliorate or prevent anger.

Women's views about dealing with anger were closely linked to the identified determinants, including the need to overcome poverty, to access skills and education, to obtain relief from the burden of household duties and care of children, to the need to have more leisure time, and to gain more support and understanding from husbands (Figure 1).

## Discussion

One in twelve Timorese women experienced IED which is characterized by explosive anger. Women with IED were more likely to live in the urban area, had higher levels of health-related disability and reported greater exposure to a triad of trauma and stresses including past and ongoing gender-related and other violence, extreme poverty, and persisting preoccupations with injustice. The qualitative data supplemented the quantitative findings by revealing women's frustrations with accessing education, employment and services, particularly health services. Husbands were implicated in women's anger, with domestic violence being associated with patriarchal values and gender inequality, exposure to war trauma, alcoholism, and mental disorder. The behaviour associated with IED amongst women led to or compounded interpersonal problems, conflict with community members and impacted adversely on the rearing of children [33].

The strengths and limitations of the study need to be considered. The sample size was large for the field and we achieved a high response rate. Mixed methods studies of this type are scant in the post-conflict mental health field and none has focused specifically on issues of explosive anger amongst women. The qualitative component proved to be invaluable in providing a more detailed account of the personal and social consequences of IED. To our knowledge, our study is the first to apply a community-based measure of IED amongst women in a culturally-distinct post-conflict setting, building on our previous results utilizing a brief screening index of explosive anger [2]. The measure showed strong concurrent validity with a gold standard clinical interview. At the same time, the study of IED is in its early phases [2], [3], [7], [34], [35] and the diagnostic criteria have been altered somewhat in the Diagnostic and Statistical Manual edition 5. The sample was restricted to two villages so that the findings may not be generalizable to the whole of Timor. As with all cross-sectional studies, there is a risk of anamnestic bias in women's accounts of previous events. Under-reporting of anger and aggression could have occurred because those reactions are regarded as socially undesirable amongst women in that culture.

While replication of our findings is necessary, the results offer some guidance in developing policy and practice in the post-conflict field, especially because there is a dearth of research focusing specifically on women's mental health in these contexts [36]–[40]. Four possible tiers of intervention warrant consideration [41]. *First,* women with disabling IED may need direct clinical interventions. Further training of local health personnel in detecting IED therefore is imperative. There is an added need however, to devise and trial interventions for IED that are sensitive to culture and gender in settings such as Timor-Leste, a task that has yet to be undertaken. *Second*, there is a need to address the sense of injustice that women experience related to the multiple human rights violations they have experienced during and subsequent to the period of conflict. Although a truth commission has been conducted in Timor-Leste [42], our data support the view that such activities may not be sufficient to address persisting feelings of injustice that remain prevalent in the community. The psychological consequences of past and ongoing human rights trauma for women in particular may be compounded by ongoing exposure to violence in the home [42]. It is important therefore to pursue programs aimed at preventing domestic violence in the aftermath of mass violence. *Third*, alleviating poverty in a manner that promotes the social status and participation of women, for example, in vocational training and employment, are priorities that should apply across all low-income, conflict-affected countries. *Finally,* a focus on child-bearing and child-rearing is important to ensure that women's frustrations with the burden of child-care do not result in the harsh treatment of their offspring, potentially creating a trans-generational pattern of abuse and trauma that will impact on the future mental health and development of the society. Parenting skills and family planning programs already being applied in other low-income countries may prove valuable if culturally adapted to Timor-Leste [43].

In considering our findings, it is vital to achieve a balance between recognizing women's anger as an expression of legitimate grievances and identifying IED as a disorder that in some instances requires mental health attention. At the most general level however, if the underlying factors perpetuating extreme and uncontrollable forms of anger are ignored, there is a risk that women, and indirectly their children, will suffer grave psychological consequences, potentially affecting progress in achieving family and social stability and the promotion of maternal and childhood mental health in conflict-affected countries such as Timor-Leste [14], [39].
